# Optimizing colonoscopy‐based colorectal cancer screening by low‐barrier, low‐threshold pretesting

**DOI:** 10.1002/ijc.70187

**Published:** 2025-10-09

**Authors:** Thomas Heisser, Rafael Cardoso, Tobias Niedermaier, Michael Hoffmeister, Hermann Brenner

**Affiliations:** ^1^ Division of Clinical Epidemiology and Aging Research German Cancer Research Center (DKFZ) Heidelberg Germany

**Keywords:** colonoscopy, colorectal cancer, fecal immunochemical testing, modelling, screening

## Abstract

‘Gateopener’ colonoscopy‐based screening is an innovative concept to better target colonoscopy to those who are most likely to benefit from it. It combines invitations to screening colonoscopy with the offer of pretesting with a single ‘gateopener’ fecal immunochemical test (FIT) which is applied with a lower positivity threshold than in conventional FIT‐based screening. We explored optimized use of this approach for reducing CRC incidence and mortality. Using COSIMO, a previously validated simulation tool, we compared outcomes of gateopener screening to those of conventional FIT‐ or colonoscopy‐based screening strategies. Gateopener screening was modelled using SENTiFIT‐FOB Gold as exemplary ‘gateopener’ FIT at various low hemoglobin cut‐offs (10, 8, 6, 4, and 3 μg/g feces). We found that Gateopener screening at cut‐offs of 6, 4, or 3 μg/g outperformed conventional screening colonoscopy in terms of CRC incidence reduction, with 16%–25%, 50%–57%, and 66%–72% more prevented cases, respectively, after 10 years. All gateopener scenarios significantly increased prevented deaths, at low cut‐offs more than doubling the numbers achieved by conventional screening colonoscopy. Compared to biennial FIT, gateopener screening prevented 7%–163% more cases, with lower cut‐offs associated with higher gains, and prevented approximately equal to significantly more (12%–21%) CRC deaths. Cut‐offs of 10 and 8 μg/g required fewer colonoscopies per prevented case and death. Gateopener screening outperforms conventional CRC screening by offering considerably stronger reduction of CRC incidence and mortality rates as well as considerably increased screening effectiveness. The feasibility of the concept should be assessed by a pilot study in real‐life practice.

## Graphical abstract

What's new?

Although colonoscopy is widely considered the gold standard for colorectal cancer (CRC) screening, it is an invasive procedure with varying adherence rates that potentially leave many high‐risk individuals unscreened. Here, the authors assessed an optimized gateopener approach to improving CRC screening efficiency using a low‐threshold fecal immunochemical test (FIT) to pre‐select individuals for colonoscopy. Simulations show that gateopener screening is associated with considerable reductions in CRC incidence and mortality. Moreover, in nearly all simulations, the approach outperformed conventional screening, regardless of hemoglobin cut‐off values. The findings warrant investigation of gateopener screening in real‐world settings.

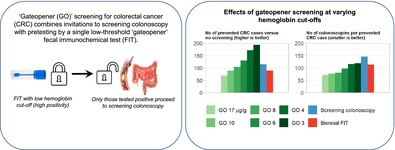

**Abbreviations**
ADN: any advanced neoplasms
ANN: any neoplasms
BLITZ: Begleitende Evaluierung innovativer Testverfahren zur Darmkrebs‐Früherkennung (English: accompanying evaluation of innovative test procedures for the early detection of colorectal cancer)
CI: confidence interval
COSIMO: colorectal cancer multistate simulation model
CRC: colorectal cancer
FIT: fecal immunochemical test
GO: gateopener
Hb: hemoglobin

## 1 INTRODUCTION

Screening colonoscopy allows for the detection and removal of precursor lesions at high sensitivity directly upon examination and is therefore, from a diagnostic perspective, regarded as the gold standard screening test for colorectal cancer (CRC).^1^ However, most screenees have no real benefit from this invasive procedure as they do not have CRC or any such precursor lesions.^2^, ^3^ Also, screening colonoscopy programs often fail to achieve adequate adherence,^4^ which implies that many of those at high CRC risk are still being missed for not using screening in the first place. Substantially higher adherence could be achieved by non‐invasive tests, such as fecal immunochemical tests (FITs),^5^ and further enhanced by optimized screening program design and invitation approaches.^6^ However, standard FITs need to be repeated annually or every other year, limiting their practicability over a life‐long timeframe.

**FIGURE 1:**
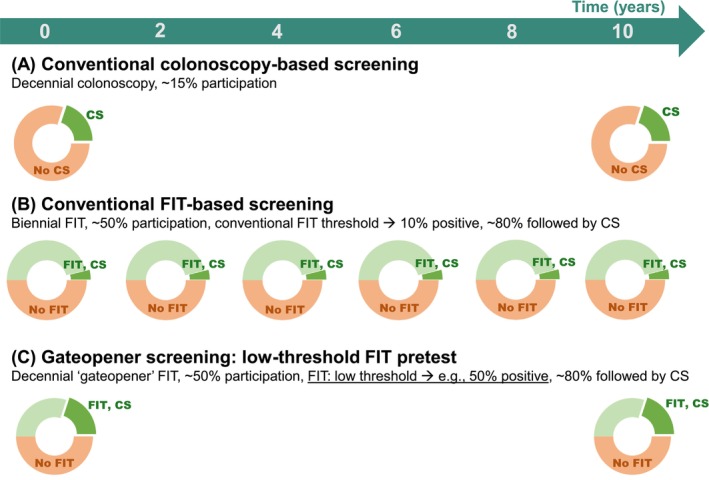
Numerical example of use of gateopener screening (C) as compared to conventional screening strategies (A, screening colonoscopy; B, biennial FIT). In the gateopener approach, instead of directly undergoing screening colonoscopy every 10 years, individuals would be invited to use a non‐invasive, low‐threshold pre‐test, such as a FIT with very low hemoglobin detection thresholds yielding higher positivities (e.g., cut‐off of 3–4 μg Hb/g, corresponding positivity: 50%, proposed in reference [7]). Only those above the threshold would be invited to follow‐up with screening colonoscopy. CS, colonoscopy; FIT, fecal immunochemical test.

We previously proposed a ‘gateopener’ approach suited to address these limiting factors in an innovative fashion, with great potential to enhance the effectiveness of CRC screening.^7^ Gateopener screening involves pre‐selecting subjects most likely to benefit, as only individuals with a positive pre‐test, such as a low threshold ‘gateopener’ FIT with low barriers to access (e.g., by directly mailing test kits) would be invited to proceed to screening colonoscopy. Gateopener pretesting with a single FIT differs from conventional biennial FIT screening in two major aspects: first, it would be offered only in 10‐year intervals, in line with recommendations on screening colonoscopy, the underlying main screening modality. Second, the hemoglobin threshold would be much lower than the one used for conventional FITs, implying significantly larger shares of positive (pre‐)test results, with the specific threshold to be used depending on the specific healthcare systems' circumstances, needs and resources. As illustrated by a numerical example in Figure 1, this approach could lead to more targeted use of the same number of colonoscopies compared to conventional untargeted screening colonoscopy. As previously demonstrated, pre‐selection by a gateopener FIT would go along with a significantly higher diagnostic yield and CRC risk reductions at the same number of conducted colonoscopies versus conventional screening colonoscopy.^7^

However, the best possible use of the gateopener approach and its performance with respect to the reduction of CRC incidence and mortality and the most efficient use of colonoscopy resources remain to be explored. For example, our initial proposal focused on the use of a gateopener FIT with hemoglobin cut‐offs of 3–4 μg/g feces yielding 50% positive test results.^7^ However, depending on available colonoscopy capacities, trained staff, and other resource constraints, higher hemoglobin cut‐offs, which might nevertheless still maintain reasonably high sensitivity for detecting advanced CRC precursor lesions, might be better suited for some health care systems, also to reduce the proportion of false‐positive gateopener FITs. Here, we sought to explore and compare the potential of various variants of gateopener screening for reducing CRC incidence and mortality and maximizing the efficient use of colonoscopy resources.

## 2 MATERIALS AND METHODS

### 2.1 Colorectal cancer multistate simulation model

To evaluate the implications of a gateopener screening approach at varying hemoglobin cut‐offs, we used the Colorectal Cancer Multistate Simulation Model (COSIMO), a validated, Markov‐based simulation tool,^8^ in a hypothetical German population. Briefly, COSIMO simulates the natural history of CRC (independent of pathogenetic pathway) based on the process of precursor lesions developing into preclinical and then clinical cancer for a predefined number of years. At the start of the simulation, certain proportions of no neoplasm, non‐advanced adenoma, advanced adenoma, and preclinical CRC are assigned to the hypothetical population. The simulation runs up to a predefined number of cycles of each year. Each year, people at each state have a certain probability (transition rate) to progress to the next state. Subjects with CRC may die from the disease, and at each state, people may experience non‐CRC death, reflecting the general background mortality from other causes. Screening can interfere with the natural history of CRC (Figure S1). People with adenoma will be moved backward to the state of no neoplasm, assuming removal of their adenoma at colonoscopy.

The model starting prevalences and transition rates are based on comprehensive data from the German national screening colonoscopy registry, a repository of all screening colonoscopies conducted in Germany with virtually complete reporting.^9^, ^10^, ^11^ CRC mortality rates by mode of detection were estimated based on the proportion of screening‐detected cases among all CRC cases in Germany combined with the overall CRC‐specific mortality rates by year after diagnosis and hazard ratios for patients detected by screening versus symptoms from Germany‐specific data sources.^12^, ^13^ COSIMO has been comprehensively validated for the German screening‐eligible population.^8^

A comprehensive documentation on the model's structure, data sources, and parameters is given in Appendix S1 and Tables S1–S3. The model source code is available from our website.^14^

### 2.2 Diagnostic performance parameters

We first derived sensitivity (the proportion of detected cases among all subjects with any adenomas or cancer) and specificity (the proportion of all subjects without adenomas or cancer correctly classified as such) parameters of SENTiFIT‐FOB Gold (Sentinel Diagnostics, Milan, Italy), a commonly used FIT test, at hemoglobin (Hb) cut‐offs of 10, 8, 6, 4, and 3 μg Hb per gram feces, respectively, which would correspond to positivity levels of approximately 20%–50%. For reference, we also derived sensitivity and specificity at the manufacturer preset cut‐off of 17 μg/g.^15^ Cut‐offs of 3–4 μg/g, corresponding to approximately 50% positivity, were originally proposed for use in the gateopener approach.^7^ Note that the lowest cut‐offs of 3–4 μg/g are above the limit of detection for SENTiFIT‐FOB Gold of 1.7 μg/g.^15^

Analyses were conducted based on 7398 participants of screening colonoscopy in Germany recruited in the BLITZ study from 2008 through 2020 who had provided pre‐colonoscopy stool samples for quantitative FITs as previously described.^15^, ^16^ Across all analyses, advanced adenomas were defined as adenomas with at least 1 of the following features: ≥1 cm in size, tubulovillous or villous components, or high‐grade dysplasia.

### 2.3 Simulations

A range of scenarios was simulated to characterize the relative effectiveness of gateopener screening at varying cut‐offs. In these scenarios, simulated cohorts of 100,000 previously unscreened women and men aged 50, 60, or 70 were assumed to be invited to undergo either:

1. conventional screening colonoscopy,
2. conventional biennial FIT screening (manufacturer preset cut‐off of 17 μg/g),
3. gateopener screening at varying hemoglobin cut‐offs (17, 10, 8, 6, 4, and 3 μg/g).

Sex‐specific baseline neoplasm prevalences for each age of screening were extracted from a previous analysis of more than 4.4 million screening colonoscopies in the German screening‐eligible population.^17^ We assumed levels of adherence reflective of those reported for the German screening‐eligible population, that is, 15% adherence for conventional screening colonoscopy (offered in 10‐year intervals) and 50% adherence for conventional biennial FIT and gateopener screening.^18^ For biennial FIT screening, a 40% drop‐out rate over time was assumed to account for the sporadic nature of adherence to FIT screening,^19^ in line with reports in the literature.^20^ Assuming 50% adherence over a 10‐year period with a 40% drop‐out results in an approximate biennial adherence rate of 20% as reported for the German screening‐eligible population by the Central Institute for Ambulatory Health Care, which was mandated with the nationwide screening program evaluation by 2019 and reports the Germany‐specific 10‐year FIT screening participation rate.^18^ We assumed that 80% of those with a positive FIT test would make use of the follow‐up colonoscopy offer, which reflects the observed compliance to diagnostic colonoscopy in real‐world screening published in a systematic review.^21^

### 2.4 Sensitivity analyses

In sensitivity analyses, to explore the impact of uncertainty related to model key parameters, all point estimates of the starting prevalences and transition rates were replaced by either the lower or upper limits of the 95% CIs. Given that the implementation of organized screening and further measures such as postal delivery of FIT test kits might imply higher adherence rates to FIT‐based screening options, we implemented a sensitivity analysis assuming 60% adherence for conventional biennial FIT and gateopener screening, and another assessing a reduced drop‐out rate of 30% for biennial FIT screening.

### 2.5 Outcomes

For each scenario, we assessed the absolute number of prevented cases and deaths as compared to no screening after 10 years of follow‐up (calculated as the difference between intervention versus no screening), the numbers of required colonoscopies and FITs, as well as the numbers of required colonoscopies and FITs per prevented cases or deaths. We then calculated and compared the corresponding relative differences for gateopener screening scenarios to those for conventional screening scenarios (screening colonoscopy or biennial FIT).

## 3 RESULTS

### 3.1 Hemoglobin cut‐offs

**FIGURE 2:**
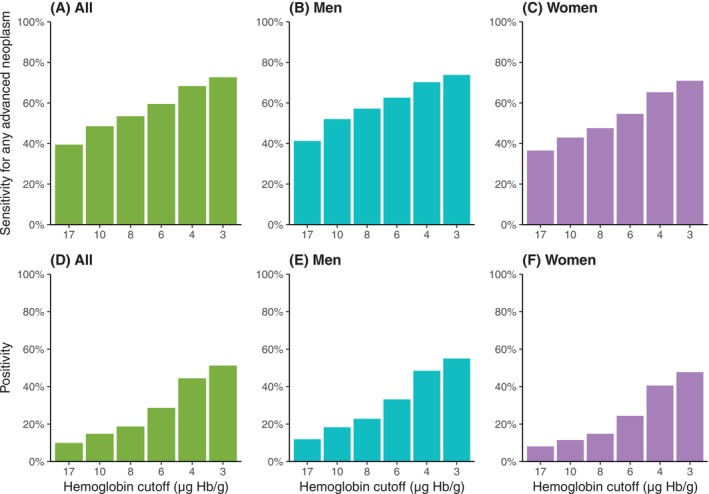
Sensitivity for any advanced neoplasm (advanced adenoma or cancer, panels A–C) and corresponding positivities (panels D–F) at varying FIT positivities.

Sensitivities for any neoplasm (ANN, any adenoma or cancer), any advanced neoplasm (ADN, any advanced adenoma or cancer), and cancer as well as specificities for no finding at varying hemoglobin cut‐offs are shown in Table 1. Sensitivities for ADN (as well as positivities) increased gradually with decreasing hemoglobin cut‐offs (Figure 2), while specificities decreased first gradually (between cut‐offs at 17 and 8 μg/g) and then rather steeply (between cut‐offs at 8 and 3 μg/g).

**TABLE 1:** Diagnostic characteristics of SENTiFIT FOB Gold at varying hemoglobin cut‐offs.

Hemoglobin cut‐off (μg Hb/g)	Positivity (%)	Sensitivity (%)			Specificity (%)
		Any neoplasm (*n* = 2115)	Any advanced neoplasm (*n* = 728)	Preclinical cancer (*n* = 54)	No advanced neoplasm (*n* = 6670)
17	10	19.6	39.4	90.7	93.3
10	15	27.4	48.5	96.3	88.9
8	19	32.1	53.4	98.1	85.1
6	29	40.8	59.5	98.1	74.7
4	44	54.5	68.3	98.1	58.2
3	51	60.4	72.7	98.1	51.1

*Note*: Analyses based on 7398 participants of screening colonoscopy in Germany from 2008 through 2020. advanced adenomas were defined as adenomas with at least 1 of the following features: ≥1 cm in size, tubulovillous or villous components, or high‐grade dysplasia.

### 3.2 Comparison to conventional screening colonoscopy

**FIGURE 3:**
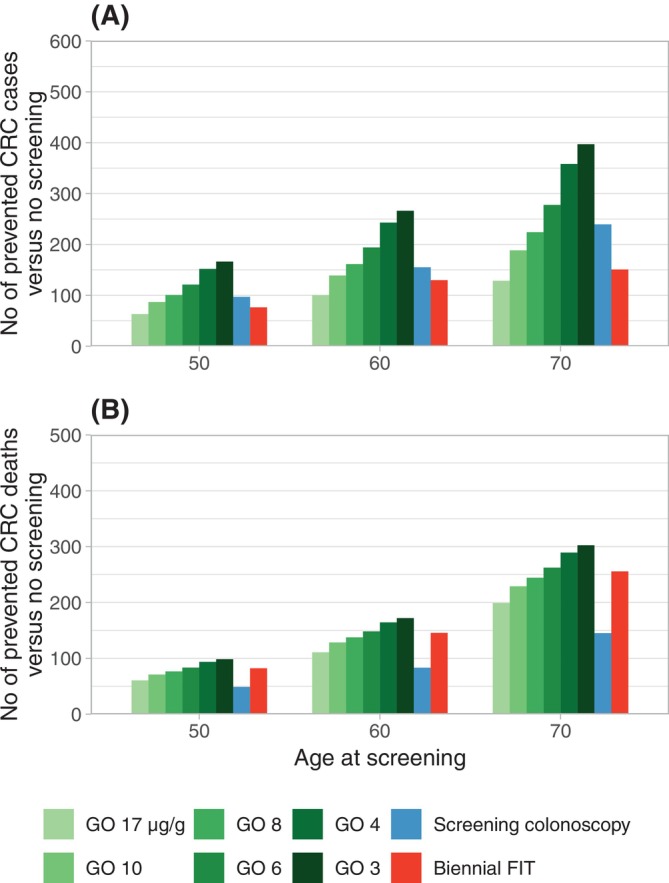
Numbers of prevented CRC cases (A) and CRC deaths (B) as compared to no screening at varying hemoglobin cut‐offs, stratified by age. CRC, colorectal cancer; FIT, fecal immunochemical testing; GO, gateopener approach.

Gateopener screening using a gateopener FIT with a cut‐off of 6, 4, or 3 μg/g outperformed screening colonoscopy in terms of CRC incidence reduction, with 16%–25%, 50%–57%, and 66%–72% more prevented cases, respectively, across all ages (Figure 3A, Table S4). Gateopener screening using a cut‐off of 17 or 10 μg/g resulted in fewer cases, and using a cut‐off of 8 μg/g resulted in approximately equal absolute numbers of prevented cases but, along with the other assessed hemoglobin thresholds, in significantly more prevented CRC deaths, with additional reductions reaching from +24% for a cut‐off of 17 μg/g up to more than twice (+109%) the number of prevented deaths for conventional screening for a cut‐off of 3 μg/g (Figure 3B). Screening using cut‐offs of 17, 10, 8, or 6 μg/g also required fewer total colonoscopies (−6% to −66%). Absolute numbers of prevented cases or deaths by gateopener screening were less marked in women versus men, but relative gains were largely consistent across sexes (Figures S2 and S3).

### 3.3 Comparison to conventional biennial FIT screening

Compared to biennial FIT screening, gateopener screening at a cut‐off of 17 μg/g prevented 15%–23% fewer CRC cases. At all other assessed cut‐offs, gateopener screening prevented more CRC cases, with relative gains ranging from 7% to 163% across all assessed ages, with lower cut‐offs associated with higher relative gains (Figure 3A, Table S4). Gateopener screening with cut‐offs of 17 and 10 μg/g resulted in fewer (−11% to −26%), with cut‐offs of 8 and 6 μg/g in approximately equal, and with cut‐offs of 4 and 3 μg/g in significantly more (+12% to +21%) prevented deaths as compared to conventional biennial FIT screening, respectively (Figure 3B). Gateopener screening using a cut‐off of 17 or 10 μg/g required 23%–52% fewer total colonoscopies, while cut‐offs of 6, 4, or 3 μg/g required 25%–26%, 79%–84%, and 102%–110% more colonoscopies, respectively. At a cut‐off of 8 μg/g, numbers of required colonoscopies were approximately comparable. The number of required FIT tests was more than halved (reduction by 54%–57%) as compared to biennial FIT screening. Results were overall consistent across men and women and assessed ages.

### 3.4 Numbers of colonoscopies per prevented case/per prevented death

**FIGURE 4:**
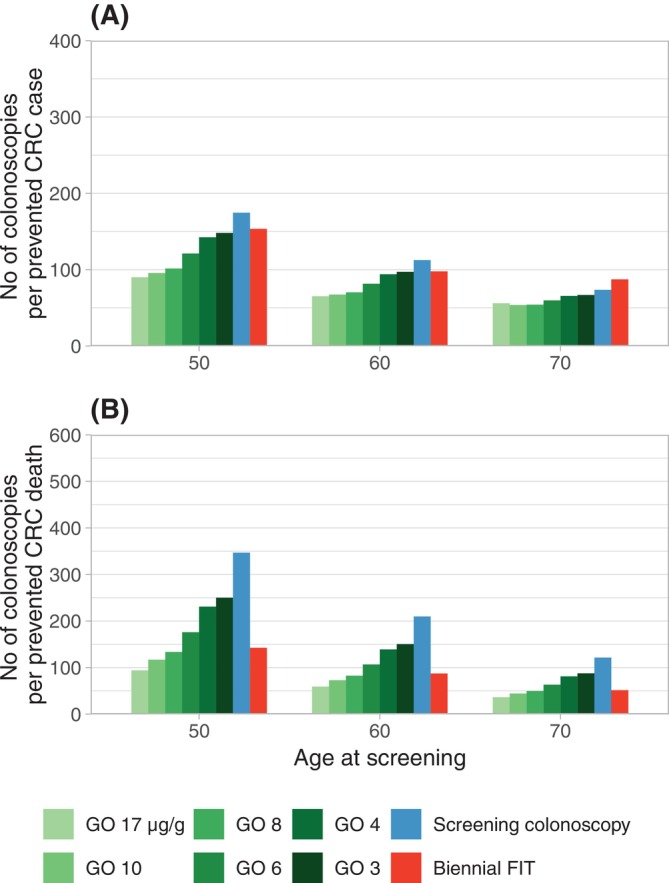
Numbers of colonoscopies per prevented CRC case (A) and CRC death (B) at varying hemoglobin cut‐offs, stratified by age. CRC, colorectal cancer; FIT, fecal immunochemical testing; GO, gateopener approach.

Across all analyses, gateopener screening with cut‐offs at 17, 10, 8, and 6 μg/g was consistently and partly substantially more efficient as compared to conventional screening strategies in terms of required numbers of colonoscopies per prevented CRC case (Figure 4A). Gateopener screening using a cut‐off of 4 or 3 μg/g was consistently more efficient than screening colonoscopy for both sexes and more efficient than conventional biennial FIT screening in men. In women, higher efficiency versus biennial FIT only manifested at higher age (Figures S4 and S5 in Appendix S2).

Gateopener screening with cut‐offs at 17, 10, or 8 μg/g required considerably fewer colonoscopies per prevented CRC death as compared to conventional screening colonoscopy, and fewer to approximately equal numbers as compared to conventional biennial FIT testing (Figure 4B). Gateopener screening with cut‐offs at 6, 4, or 3 μg/g was still consistently more efficient than screening colonoscopy but required more colonoscopies per prevented death than biennial FITs. For both measures (numbers per prevented case/deaths), differences across varying gateopener cut‐offs tended to become smaller with increasing age at screening.

### 3.5 Sensitivity analyses

Sensitivity analyses using upper and lower limits of 95% CI of starting prevalences and annual transition rates yielded comparable patterns to the base case scenarios (Figures S6–S9 in Appendix S2). Effects of gateopener screening were consistent with varying assumptions on adherence to FIT testing (Figures S10–S13 in Appendix S2).

## 4 DISCUSSION

Conventional screening colonoscopy is a relatively burdensome and invasive procedure with generally low adherence rates.^18^ Only a few individuals benefit from this resource‐intensive procedure, making it rather inefficient on a population level. According to previous estimates, approximately 200 and 400 screen‐eligible men and women aged 50 years, respectively, would need to be screened to detect a single case of cancer.^7^

Adherence to conventional fecal‐test‐based screening is higher, owing to its non‐invasive nature and more convenient conduct. However, when used at manufacturer preset hemoglobin thresholds, sensitivity is high for preclinical cancers but suboptimal for precursor lesions (e.g., at the cutoff of 17 μg/g, sensitivity for any neoplasm and any advanced neoplasm is 20% and 39%, respectively, while it is 91% for preclinical CRC).^15^, ^22^ FIT screening therefore needs to be repeated annually or every other year, requiring substantial resources and impairing its practicability over a lifelong timeframe. In effect, a considerable proportion of users follows suboptimal sporadic adherence patterns, and adherence is highest in the first screening round.^19^, ^23^, ^24^

These limitations imply that conventional screening approaches alone are unlikely to mount a sufficient response to the predicted strong increase of the future CRC burden.^25^, ^26^, ^27^, ^28^ Gateopener screening is a strong candidate to address this gap. It unites the advantages of FIT‐based screening, that is, high adherence and low barriers of use, with those of primary screening colonoscopy, that is, detection of precursor lesions with high sensitivity. As only a single FIT is needed every 10 years, this should simplify personal invitations and allow for postal provision, which could lead to even higher adherence.^6^ Another key feature of gateopener screening is the ability to direct colonoscopy capacities to those most likely to benefit while maintaining or likely improving overall screening outcomes.

In our study, we illustrated several possibilities to implement gateopener screening. From a public health perspective, the chosen hemoglobin cut‐off for the gateopener FITs reflects a continuum: on the end with low hemoglobin thresholds, screening outcomes, that is, numbers of prevented cases and deaths are maximized, at the cost of lower screening efficiency (higher share of false‐positive tests and higher numbers needed to screen). At the other end, with high hemoglobin thresholds, screening efficiency is highest (lower numbers needed to screen), at the cost of fewer overall prevented cases and deaths. However, in virtually all simulations, regardless of the eventually chosen cut‐off, gateopener screening outperformed conventional screening on either or both metrics.

The comparably high false‐positive rate of low cut‐off gateopener screening should not be used as an argument against this approach. For conventional primary screening colonoscopy, 70%–80% of individuals undergo the procedure without any finding, and close to 90% have no advanced finding.^29^ More than 2000 and 3500 colonoscopies in 50‐year‐old men and women, respectively, were estimated to be needed to detect a single case of cancer in those who would hypothetically undergo screening colonoscopy but were prevented from doing so due to negative pretesting with a gateopener FIT using very low cut‐offs of 3–4 μg/g.^7^

Combined, this suggests that most individuals do not benefit from conventional screening colonoscopy, and gateopener screening could possibly significantly improve screening efficiency by preventing a large share of unnecessary colonoscopies despite high false‐positive rates. Clear patient communication on the advantages and specifics of gateopener screening will be key, ideally in a well‐formulated invitation letter. Smart program design involving such inviting letters as well as postal provision of FITs would likely substantially improve the adherence and acceptance.^6^ The potential and practical implications of the gateopener approach in a real‐life screening setting should be assessed by a pilot study.

Of note, in our simulations, a high procedural quality of colonoscopies is assumed as COSIMO is based on data from the German national screening colonoscopy registry, and certification to conduct screening colonoscopy in Germany is tightly regulated and subject to rigorous quality control. Incidence of post‐colonoscopy CRC is significantly lower when colonoscopists undergo an accreditation process involving strict, standardized performance targets.^30^ Although our study supports further exploration of the potential of the gateopener concept, the fact that a high procedural quality of the colonoscopy remains a paramount prerequisite should be kept in mind.

## 5 LIMITATIONS

Specific limitations of COSIMO have been described previously.^7^, ^8^ Briefly, major limitations concern simplifying model assumptions and uncertainties related to input parameters. For instance, true adenoma miss rates at colonoscopy are unknown, and we used the best available evidence from a systematic review and meta‐analysis for approximation.^1^ Furthermore, as subsite‐specific data was not available in the registry data used to inform COSIMO model parameters,^8^ no stratified analysis for distal versus proximal CRC was possible, which would have allowed for comparisons to screening sigmoidoscopy, another broadly established endoscopic screening intervention. As well, the difference in times between a positive stool blood test and the performance of colonoscopy is not modelled, which may impact the results.^31^ The follow‐up period was limited to 10 years since the modelled scenarios implement assumptions on adherence. Modelling beyond a 10‐year horizon would require taking additional and strong assumptions on repeated use of gateopener screening, thereby further complicating analyses and interpretation of results.

In addition, simulations were conducted for a screen‐eligible population in Germany (for which COSIMO was calibrated and validated^8^), and baseline prevalences as well as adenoma and cancer incidence rates might vary for other populations with varying distributions of CRC risk factors. Also, input adherence patterns were derived from German data sources, and adherence will likely vary across countries and healthcare systems. However, in our study, key outcome parameters should be interpreted relative to the measured effectiveness of conventional screening approaches, and it appears plausible that the relative effectiveness of gateopener screening will be consistent across populations. A real‐life pilot study is needed to assess the plausibility of adherence assumptions as well as the overall feasibility of the gateopener concept.

Further research should also include comparative cost‐effectiveness and cost–benefit calculations on the effect of gateopener screening as compared to conventional strategies to more fully assess the viability of gateopener screening for health care systems. Such analyses should also address the cost increase associated with a potential postal delivery of gateopener FIT test kits, as well as the likely cost savings due to improved CRC prevention and early diagnosis.

## 6 CONCLUSION

Gateopener screening is an innovative concept for the prevention and early detection of CRC. The approach involves low barrier, low threshold non‐invasive pretesting using a single ‘gateopener’ FIT followed up by screening colonoscopy for those with positive pre‐test results. This ‘gateopener FIT’ may be adjusted by modifying hemoglobin cut‐offs indicating when such pre‐test will be considered positive, whereby relatively lower cut‐offs will imply higher numbers of prevented CRC cases and deaths, and relatively higher cut‐offs will imply higher screening effectiveness (i.e., fewer colonoscopies to detect one individual with an advanced finding). This study illustrates that gateopener screening at varying cut‐offs will outperform conventional screening approaches by offering considerably stronger reduction of CRC incidence and mortality rates as well as considerably increased screening effectiveness. The feasibility of the concept should be assessed by a pilot study in real‐life practice.

## AUTHOR CONTRIBUTIONS

**Thomas Heisser:** Conceptualization; writing – original draft; methodology; visualization; writing – review and editing; formal analysis. **Rafael Cardoso:** Writing – review and editing. **Tobias Niedermaier:** Writing – review and editing. **Michael Hoffmeister:** Writing – review and editing. **Hermann Brenner:** Conceptualization; supervision; writing – review and editing; funding acquisition; methodology.

## FUNDING INFORMATION

Financial support for this study was provided in part by grants from the German Federal Ministry of Education and Research (grant numbers 01GL1712 and 01KD2104A) and from the German Cancer Aid (70114735). The funding agreements ensured the authors' independence in designing the study, interpreting the data, writing, and publishing the report.

## CONFLICT OF INTEREST STATEMENT

The authors declare that they have no conflict of interest.

## ETHICS STATEMENT

The BLITZ study was approved by the ethics committees of Heidelberg University (178/2005) and the state medical chambers of Baden‐Württemberg (M118‐05‐f), Saarland (217/13), Rhineland Palatinate (837.047.06[5145]), and Hesse (MC 254/2007). All participants in BLITZ provided written informed consent.

## Supporting information


**APPENDIX S1:** Supporting information.

## Data Availability

All analyses relevant to the study are included in the article or uploaded as supplementary information. The model source code is freely available from the DKFZ website (https://www.dkfz.de/de/klinepi/download/index.html). Further information is available from the corresponding author upon request.
